# Central Projections of Antennal and Labial Palp Sensory Neurons in the Migratory Armyworm *Mythimna separata*

**DOI:** 10.3389/fncel.2017.00370

**Published:** 2017-11-21

**Authors:** Bai-Wei Ma, Xin-Cheng Zhao, Bente G. Berg, Gui-Ying Xie, Qing-Bo Tang, Gui-Rong Wang

**Affiliations:** ^1^Department of Entomology, College of Plant Protection, Henan Agricultural University, Zhengzhou, China; ^2^Department of Psychology, Norwegian University of Science and Technology, Trondheim, Norway; ^3^Department of Pesticide, College of Plant Protection, Henan Agricultural University, Zhengzhou, China; ^4^State Key Laboratory for Biology of Plant Disease and Insect Pests, Institute of Plant Protection, Chinese Academy of Agricultural Sciences, Beijing, China

**Keywords:** *Mythimna separata*, antenna, labial-palp pit organ, olfaction, mechanosensation, central projections, central nervous system

## Abstract

The oriental armyworm, *Mythimna separata* (Walker), is a polyphagous, migratory pest relying on olfactory cues to find mates, locate nectar, and guide long-distance flight behavior. In the present study, a combination of neuroanatomical techniques were utilized on this species, including backfills, confocal microscopy, and three-dimensional reconstructions, to trace the central projections of sensory neurons from the antenna and the labial pit organ, respectively. As previously shown, the axons of the labial sensory neurons project via the ipsilateral labial nerve and terminate in three main areas of the central nervous system: (1) the labial-palp pit organ glomerulus of each antennal lobe, (2) the gnathal ganglion, and (3) the prothoracic ganglion of the ventral nerve cord. Similarly, the antennal sensory axons project to multiple areas of the central nervous system. The ipsilateral antennal nerve targets mainly the antennal lobe, the antennal mechanosensory and motor center, and the prothoracic and mesothoracic ganglia. Specific staining experiments including dye application to each of the three antennal segments indicate that the antennal lobe receives input from flagellar olfactory neurons exclusively, while the antennal mechanosensory and motor center is innervated by mechanosensory neurons from the whole antenna, comprising the flagellum, pedicle, and scape. The terminals in the mechanosensory and motor center are organized in segregated zones relating to the origin of neurons. The flagellar mechanosensory axons target anterior zones, while the pedicular and scapal axons terminate in posterior zones. In the ventral nerve cord, the processes from the antennal sensory neurons terminate in the motor area of the thoracic ganglia, suggesting a close connection with motor neurons. Taken together, the numerous neuropils innervated by axons both from the antenna and labial palp indicate the multiple roles these sensory organs serve in insect behavior.

## Introduction

In moths, the antennae and the labial palps represent two prominent appendages for sensory input, involved in multiple behaviors, such as foraging, orientation, feeding, mating, and flight (Schneider, 1964; Guerenstein and Hildebrand, 2008; Krishnan and Sane, 2015). The labial-palp pit organ (LPO), located on the tip of each labial palp, possesses olfactory sensilla specialized for sensing carbon dioxide (CO_2_, Guerenstein and Hildebrand, 2008). Previous findings, in both the pyralid moth, *Cactoblastis cactorum*, and sphingid moth, *Manduca sexta*, have shown that sensing of CO_2_ mediates oviposition and nectar feeding behaviors (Stange, 1997; Guerenstein et al., 2004). Axons of LPO sensory neurons project via the labial nerve to three main regions of the central nervous system: (1) to the LPO glomerulus (LPOG) in each antennal lobe, the primary olfactory center in the insect brain, (2) to the gnathal ganglion, and (3) to the ventral nerve cord (Bogner et al., 1986; Kent et al., 1986; Zhao et al., 2013). The antennal axons, on the other hand, target (1) all glomeruli of the ipsilateral antennal lobe except for the LPOG, (2) the antennal mechanosensory and motor center (AMMC), (3) the gnathal ganglion, and (4) the ventral nerve cord (Xie et al., 2016). Whereas the LPO houses sensory neurons tuned to one odor cue only, i.e., CO_2_, the antennal neurons are tuned not only to a large amount of odor stimuli but to several sensory modalities including taste, mechano-sensation, humidity, and temperature as well (Schneider, 1964; Altner and Loftus, 1985; Nishikawa et al., 1995; Jørgensen et al., 2006; Guerenstein and Hildebrand, 2008; Popescu et al., 2013; Enjin et al., 2016; Frank et al., 2017). The numerous odor neurons are housed inside various types of olfactory sensilla situated on the flagellum, such as trichoid sensilla, basiconic sensilla, and coeloconic sensilla (Schneider, 1964; Keil, 1999). These olfactory sensilla, being involved in detecting a variety of pheromones and host volatiles (de Bruyne et al., 2001; Christensen and Hildebrand, 2002), project their sensory axons exclusively into the antennal-lobe glomeruli. Furthermore, olfactory sensory neurons expressing a distinct odorant receptor type project to one or two glomeruli (Gao et al., 2000; Vosshall et al., 2000; reviewed by Christensen and Hildebrand, 2002).

In the moths *Heliothis virescens* and *Spodoptera littoralis*, antennal gustatory neurons, housed in sensilla chaetica, are reported to project into a subregion of the gnathal ganglion named the gustatory area of the moth brain (Jørgensen et al., 2006; Popescu et al., 2013). No thermo- or hygroreceptor neurons have so far been identified on the moth antenna. In the cockroach *Periplaneta americana* and the fruit fly *Drosophila melanogaster*, however, such neurons are encapsulated inside grooved hairs and pegs and project to glomeruli in the ventro-posterior part of the antennal lobe (Nishikawa et al., 1995; Enjin et al., 2016). Antennal mechanosensory neurons are located in a variety of sensilla, including Böhm bristles, sensilla of Johnston’s organ, and the above-mentioned s. chaetica, which are located on the flagellum. The mechanosensory neurons housed by s. chaetica, which are co-localized with chemosensory neurons, have large-diameter fibers targeting the AMMC (Jørgensen et al., 2006). Böhm bristles are located on the scape and pedicel, at the joint of scape-head and scape-pedicel, respectively. They detect the position of the antennae and control the insect steering during flight (Sane et al., 2007; Krishnan et al., 2012). Axons originating from Böhm bristles terminate in the AMMC (Krishnan et al., 2012). Johnston’s organ, which is located at the inner surface of the pedicel, houses chordotonal neurons sensing a wide range of airflow vibrations or gravity (Yorozu et al., 2009; Dieudonné et al., 2014). Axons of Johnston’s organ neurons (JONs) in the *D. melanogaster*, and honey bee, *Apis mellifera*, are found to be broadly distributed in several areas, including the AMMC (which is called the dorsal lobe in *A. mellifera*), dorsal part of the gnathal ganglion, and ventro-posterior protocerebrum (Kamikouchi et al., 2006; Ai et al., 2007). Overall, antennal neurons housed inside morphologically specific sensillum categories detect distinct sensory modalities and project to different areas in the central nervous system, allowing the behaving insect to process the input from the different stimuli simultaneously.

The oriental armyworm moth, *Mythimna separata* (Walker) (Lepidoptera: Noctuidae) is a migratory, polyphagous pest, feeding on numerous plants in worldwide areas, including wheat, rice, corn, cotton, beans, as well as many vegetables (Lin, 1990; Jiang et al., 2014). Like other moth species, *My. separata* relies on olfaction for locating food and mates (He et al., 2017). In addition, *My. separata* is a migratory species, displacing seasonally over long distances (Chen et al., 1989; Feng et al., 2008; Zhao et al., 2009; Zhang et al., 2013). The multiple categories of antennal sensilla sensing different stimulus modalities simultaneously are probably an essential element for guiding this kind of behavior. However, the detailed projection pathways of the antennal sensory neurons in the central nervous system of *My. separata* are unknown.

*Mythimna separata* possesses a pair of typical filiform antennae, bearing numerous sensilla of different morphological types. On the flagellum, typical categories include s. chaetica, s. trichoidea, s. basiconica, and s. coeloconica (Chang et al., 2015). On the two basal segments, the scape and pedicel, there are many Böhm bristles (Chang et al., 2015). In addition to the antennal sensilla, Johnston’s organ constitutes a particular structure housing numerous sensory neurons. Like other noctuid moths, *My. separata* possesses a large number of LPO sensilla as well (Dong et al., 2014). In the present study, the central projections from these sensory organs were investigated by using fluorescent staining combined with confocal microscopy and digital imaging reconstructions. The results provide a comprehensive map of the antennal and labial sensory neurons in the central nervous system of *My. separata* and will ultimately aid understanding of the neuronal processes controlling multiple behaviors.

## Materials and Methods

### Insect Rearing

Female and male adults of *My. separata* were used for the experiments. Larvae were reared on an artificial diet in the laboratory under the conditions of 27°C, 70% relative humidity, and a 16/8 h light/dark cycle. Adults were fed a 10% honey solution.

### Scanning Electron Microscopy

An environmental scanning electron microscope was used to observe the antennal sensilla. The antennae were fixed in 2.5% glutaraldehyde and then dehydrated with graded ethanol series (50, 70, 90, 96, and 100%). After being mounted on a stub, the antennae were air-dried and gold-coated with sputter-coating before being examined with the electron microscope.

### Staining of Antennal Sensory Neurons

The antennal sensory neurons were mapped by utilizing different staining experiments including (1) dye applied to the base of the scape, visualizing all sensory neurons, (2) dye applied to the base of the pedicel, visualizing all neurons except for those on the scape, (3) dye applied to the base of the flagellum, visualizing the flagellar neurons exclusively, (4) dye applied to cut s. trichoidea on the flagellum, visualizing a selection of olfactory afferents, (5) dye applied to cut sensilla forming the bristle of the pedicel, plus sensilla of the Johnston’s organ, (6) dye applied to sensilla of the bristle on the scape. In all the staining experiments, the adult insect was fixed in a plastic tube with dental wax so that the head was exposed. In the first three experiments, the antenna was cut at the base of the scape, pedicel, and flagellum, respectively, as explained above. Crystals of the fluorescent dye, tetramethylrhodamine dextran (Micro-Ruby, Molecular Probes; Invitrogen, Eugene, OR, United States) were applied at the cut surface by using a needle. In the last three experiments mentioned, dye crystals were applied onto cut sensilla in the relevant regions. After all kinds of staining, the animal was placed in the fridge with a moist filter paper overnight allowing transportation of the dye in the sensory axons. On the second day, the brain and ventral nerve cord was dissected out in Ringer’s saline, and then fixed for 1 h in 4% paraformaldehyde in 0.1 M phosphate-buffered saline (PBS, pH 7.4). Following dehydration with an ascending ethanol series, the preparations were cleared in methylsalicylate, and mounted in Permount in perforated aluminum slides with two glass coverslips. In addition to the staining experiments described above, one experiment of retrograde neural labeling was performed. Here, dye was applied onto the cut end of the antennal nerve close to its entrance into the antennal lobe, in order to visualize the sensory neurons of the Johnston’s organ.

### Staining of Sensory Neurons Projecting from the LPO

The sensory neurons originating in the LPO were labeled by dye being applied onto the cut tip of the most peripheral segment of one labial palp. The following treatment of the stained preparations was similar to that described in the section above.

### Immunocytochemistry with Synapsin

For visualizing central neuropil structures in the central nervous system of the moth, some of the mass-stained preparations were labeled by means of synapsin immunocytochemistry as well. After being fixed in 4% paraformaldehyde in PBS and rinsed, as described above, the brain and ventral nerve cord was pre-incubated with 5% NGS (Sigma, St. Louis, MO, United States) in 0.1M PBS containing 0.5% Triton X-100 (PBST; 0.1 M, pH 7.4) overnight at 4°C. The primary antibody SYNORF1 (Developmental Studies Hybridoma Bank, University of Iowa), at a concentration of 1:100 (with 5% NGS in PBST) was then applied, and the preparation was kept at 4°C for 5 days. Following rinse in PBS 6 × 20 min, the brain and ventral nerve cord was incubated in the secondary antibody, Cy2-conjugated anti-mouse (Invitrogen, Eugene, OR; dilution 1:300 with 1% NGS in PBST), for 3 days at 4°C. Finally, the preparation was washed 6 × 20 min in PBS, dehydrated with graded ethanol series (50, 70, 90, 96, each 10 min, and 100% 2 × 10 min), cleared in methylsalicylate, and mounted in Permount, as described above.

### Image Acquisition and Analysis

A confocal laser scanning microscope (LSM 780, META Zeiss, Jena, Germany) was used to obtain images of the sensory neurons and the neuropils. Two objectives were utilized, a Plan-Neofluar 10 × /0.3 for large-scale images and a 20 × /0.5l for higher resolution. The Cy2, displaying brain structures, was excited by an argon laser of 488-nm line and the Micro-Ruby, displaying the stained neurons, by a HeNe laser 543-nm line. The resolution of the confocal images was obtained at 1024 × 1024 voxels with intervals of 3 or 4 μm.

Identified neuropils within the brain and ventral nerve cord were reconstructed by using the Amira software (Amira 5.3, Visage Imaging, Fürth, Germany). The stained axons and neuronal processes were reconstructed manually as described previously by means of the skeleton tool of Amira (Zhao et al., 2016).

## Results

### Morphological Features of Sensilla and Neurons Confined to the Antenna

**FIGURE 1:**
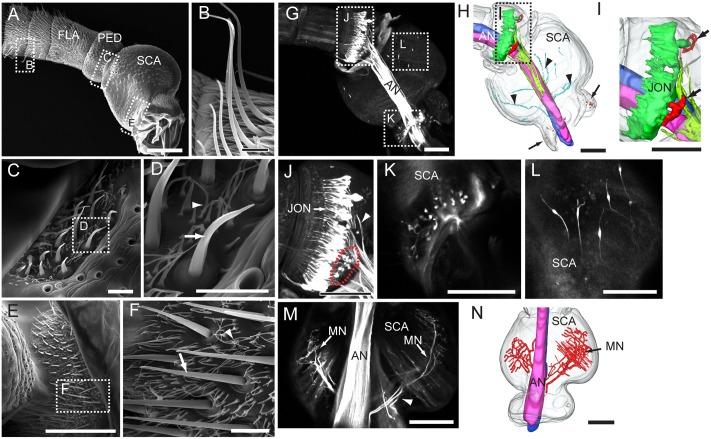
Morphological properties of the antenna, including its segments, sensilla, and sensory neurons. **(A)** Proximal part of the antenna with all three segments: scape (SCA), pedicel (PED), and four annuli of the flagellum (FLA). **(B)** Sensilla trichoidea on the flagellum. **(C,D)** Böhm bristles on the pedicel. **(E,F)** Böhm bristles on the scape. **(G)** Confocal image of the antennal nerve (AN) and antennal sensory neurons of the pedicel and scape, visualized by retrograde staining. **(H)** Three-dimensional reconstruction showing the stained AN and antennal sensory neurons of the pedicel and scape. Arrows indicate the sensory neurons of Böhm bristles on the scape. Arrowheads indicate axons of sensory neurons on the inner cuticular surface of the scape. **(I)** Three-dimensional reconstruction showing neurons of Johnston’s organ (JONs) and sensory neurons of Böhm bristles on the pedicel (indicated by arrows). **(J)** Confocal image of JONs and sensory neurons of Böhm bristles on the pedicel (indicated by red dotted circle). Arrowhead indicates axons of JONs. **(K)** Confocal image of sensory neurons of Böhm bristles on the scape. **(L)** Confocal image of sensory neurons on the inner cuticular surface of the cuticle. **(M)** Confocal image of motor neurons (MN) linked to intrinsic muscles of the scape. **(N)** Three-dimensional reconstruction showing the motor neurons. Scale bars, **(A,G–N)** 100 μm. **(B–F)** 10 μm.

The images from electron microscopy demonstrated that the antennae of *My. separata* are covered by morphologically different categories of sensilla (**Figure 1**; Chang et al., 2015). The flagellum is the site for several types of sensilla including s. trichoidea, s. basiconica, s. coeloconica, and s. styloconica. The most abundant category is the s. trichoidea appearing as long hairs (**Figures 1A,B**) On the pedicel and scape, the Böhm bristles are gathered in clusters at the joints of the pedicel-scape and the scape-head, respectively (**Figures 1A,C,E**). There are two clusters of Böhm bristles on the pedicel and two on the scape, positioned opposite to each other (**Figures 1C,E**). The number of bristles on the scape is relatively large. Böhm bristles comprise short s. chaetica (indicated by arrows in **Figures 1D,F**) surrounded by many short seta (indicated by arrowheads in **Figures 1D,F**).

Among 12 attempted trials including dye applied to the proximal antennal nerve (AN), for labeling the peripheral part of the antennal sensory neuron, eight were successful. The retrograde staining revealed that the AN is organized into two sub-bundles formed mainly by axons of flagellar sensory neurons (**Figures 1G,H**). At the proximal part of the pedicel, there is a large number of bipolar neurons confined to the Johnston’s organ (**Figures 1G,H,J**). The dendrites of these sensory neurons extend into the distal part of pedicel (**Figure 1J**) whereas their axons join the AN from a surrounding position (**Figures 1H,J**). The Böhm bristles, which were found both on the scape and pedicel, encapsulate sensory neurons extending their dendrites under the cuticular protrusion (scape: **Figures 1G,K**; pedicel: **Figures 1G,J**, indicated by red dotted circle line). Clusters of these sensory neurons are located opposite to each other, two on the scape and two on the pedicel (indicated by arrows in **Figures 1H,I**). In addition to sensory neurons of Böhm bristles, a few bipolar neurons were observed in the proximal part of the scape (**Figures 1G,H,L**). These neurons resemble those of the Johnston’s organ by extending their dendrites toward the cuticle and having axons joining the AN (**Figure 1H** indicated by arrowheads). The AN also includes some motor neurons which innervate intrinsic muscles of the scape (**Figures 1M,N**).

### Overview of the Antennal-Axon Terminals in the Central Nervous System

**FIGURE 2:**
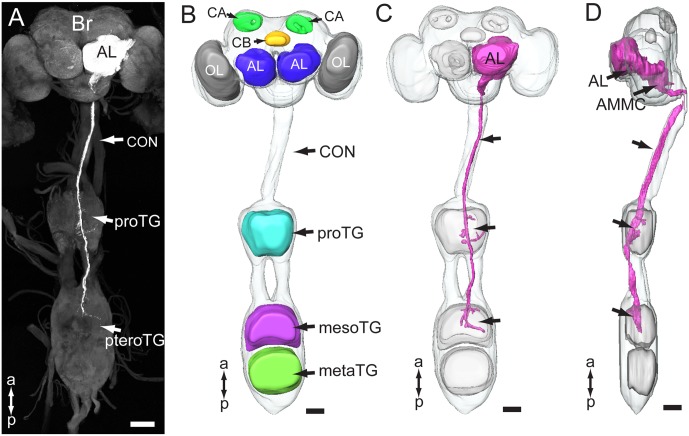
Gross projection pathway of the antennal sensory neurons in the central nervous system of *My. separata*. **(A)** Confocal image of antennal axons terminating in the brain and ventral nerve cord. **(B)** Three-dimensional reconstruction showing prominent structures of the central nervous system. The brain and thoracic ganglia are linked by the long connective (CON). Brain neuropils include the optic lobes (OL), antennal lobes (AL), central body (CB), and calyx (CA). Ventral-cord neuropils include the thoracic ganglia, prothoracic ganglion (proTG), mesothoracic ganglion (mesoTG), and metathoracic ganglion (metaTG). The mesoTG and metaTG are fused forming the pterothoracic ganglion (pteroTG). **(C,D)** Three-dimensional reconstruction showing the overall projection pathway of the antennal sensory neurons in the central nervous system (frontal and lateral view, respectively). AMMC, antennal mechanosensory and motor center; Br, brain. a, anterior; p, posterior. Scale bars, 100 μm.

The gross innervation pattern of antennal sensory neurons in the central nervous system of *My. separata* was mapped from confocal images of 10 successfully stained preparations (among 21 trials), in which dye was applied to the base of the scape. The axons of all antennal afferents project via the AN to the ipsilateral side of the central nervous system, including the antennal lobe (AL), lateral protocerebrum, AMMC, gnathal ganglion, plus the prothoracic and mesothoracic ganglion of the ventral nerve cord (**Figures 2A,C,D** and **Table 1**). Two kinds of additional staining experiments including dye applied to the base of the pedicel (20 trials) and flagellum (24 trials) resulted in 12 and 14 successfully stained preparations, respectively. All these preparations showed labeled projections in the regions mentioned above, including the AL, lateral protocerebrum, AMMC, gnathal ganglion, and ventral nerve cord. However, whereas the specific innervation patterns in the AL and the lateral protocerebrum were similar across preparations stained from the three different antennal segments, they differed in the remaining target regions (**Table 1**). Males and females displayed identical projection patterns.

**Table 1:** Projection patterns of different assemblies of antennal sensory neurons in the central nervous system of *Mythimna separata*.

			Target areas of neural processes					
			
Location of dye applying	Stained neurons	Location of cell body	PR	AL	AMMC	GNG	proTG	mesoTG
Scapal base	Antennal SN	Antennal sensilla	Lateral PR	All glomeruli but not LPOG	Zones 1 – 8	Medial GNG	Medial and lateral proTG	Medial and lateral mesoTG
	A putative CN	Ventro-lateral to ES	Not resolved	no	Ventral AMMC	no	no	no
	Antennal MN	Lateral to AMMC	no	no	Zone 9	no	no	no
Scapal Böhm bristles	Böhm bristle SN	Böhm bristle sensilla	no	no	Zones 4,5,7 and 8	no	Medial and lateral proTG	Medial and lateral mesoTG
Pedicellar base	Pedicelar and flagellar SN	Antennal sensilla	Lateral PR	All glomeruli but not LPOG	Zones 1 – 8	Medial GNG	Medial and lateral proTG	Medial and lateral mesoTG
Pedicellar Böhm bristles	Böhm bristle SN	Böhm bristle sensilla	no	no	Zones 4, 5, 7 and 8	no	Medial and lateral proTG	Medial and lateral mesoTG
Johnston’s organ	JON	Pedicel	no	no	Zones 4 – 8	no	Medial and lateral proTG	Medial and lateral mesoTG
Flagellum base	Flagellar SN	Flagellar sensilla	Lateral PR	All glomeruli but not LPOG	Zones 1 – 4, 7 and 8	Medial GNG	Medial and lateral proTG	Medial and lateral mesoTG
Sensilla trichoid	Olfactory SN	Sensilla trichoid	no	Many glomeruli	not	no	no	no
LPO base	LPO SN	LPO sensilla	no	LPOG	not	Medial GNG	Antero-ventral proTG	no


*AL, antennal lobe; AMMC, antennal mechanosensoy and motor center; CN, centrifugal neuron; ES, esophagus; GNG, gnathal ganglion; JON, Johnston’s organ neuron; LPO, labial-palp pit organ; LPOG, labial-palp pit organ glomerulus; mesoTG, mesothoracic ganglion; MN, motor neuron; PR, protocerebrum; proTG, prothoracic ganglion; SN, sensory neuron.*

### Central Projection Patterns of Antennal Sensory Neurons in the AL and the Protocerebrum

**FIGURE 3:**
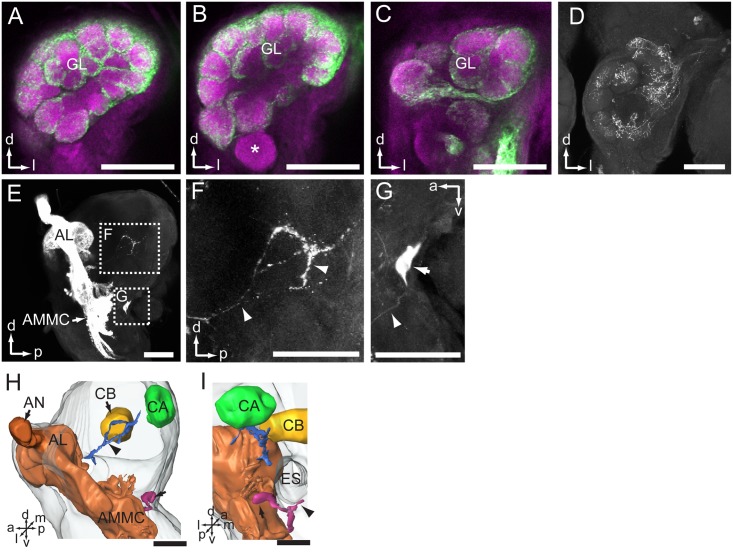
Central projections of antennal sensory neurons in the antennal lobe (AL) and lateral protocerebrum of the brain. **(A–C)** Confocal images of antennal sensory neurons (green) targeting the core of AL glomeruli (GL, magenta). Asterisk in **(B)** indicates one large GL devoid of antennal axons. **(D)** Projections of sensory neurons located on the flagellum, visualized via selective staining of sensilla trichoidea. **(E)** Sagittal view of antennal sensory projections in the brain, targeting the AL, AMMC, and the lateral protocerebrum. **(F)** Enlarged image of the stained axons in the lateral protocerebrum. Arrowheads indicate neuron processes. **(G)** One big soma located posteriorly in the brain, under the esophagus (ES), was stained (arrow). The arrowhead indicates the neurite. **(H,I)** Three-dimensional reconstruction showing projections of antennal sensory neurons in the brain (lateral and posterior view, respectively). The arrow indicates the cell body. The arrowhead indicates the processes. **(I)** AMMC, antennal mechanosensory and motor center; AN, antennal nerve; CA, calyx; CB, central body. a, anterior; d, dorsal; l, lateral; m, medial; p, posterior; v, ventral. Scale bars, 100 μm.

The most prominent target area of the antennal afferents was the AL (**Figures 2**, **3A–C**). Here, in the primary olfactory center, the sensory axons form a characteristic pattern consisting of hollow spheres, established by terminals innervating the periphery of the AL glomeruli (**Figures 3A–C**). All glomeruli receive input from the antennal axons, except for one, which is located most ventrally in the AL (indicated by asterisk in **Figure 3B**). The projection pattern in the AL was identical in all successfully stained preparations no matter if dye had been applied to the scape, pedicel, or flagellum (**Table 1**). Specific staining of selected s. trichoidea, situated on the flagellum, showed labeled axons innervating the AL glomeruli exclusively (**Figure 3D** and **Table 1**).

In addition to the numerous axons targeting the AL, a few stained fibers projected to the ipsilateral protocerebrum of the brain (**Figures 3E,F,H,I** and **Table 1**). This region, which is positioned laterally to the central body and anteriorly of the mushroom body calyx, was innervated by labeled processes projecting via the posterior AL (**Figure 3H** indicated by an arrowhead). Again, the projection pattern appeared similar in all stained preparations.

In addition to the terminals of sensory neurons, a big soma located at the posterior border of brain, on the ventrolateral side of esophagus, was stained (**Figures 3E,G**). Its primary neurite bifurcated; one branch targeted the AMMC and the other projected ventrally of the esophagus to the contralateral side (**Figure 3I**, indicated by an arrowhead). It may be a centrifugal neuron innervating sensilla located on the flagellum. Unfortunately, the terminals of these prominent neural processes could not be traced owing to the weak staining.

### Projection Pattern of Antennal Sensory Neurons in the AMMC

**FIGURE 4:**
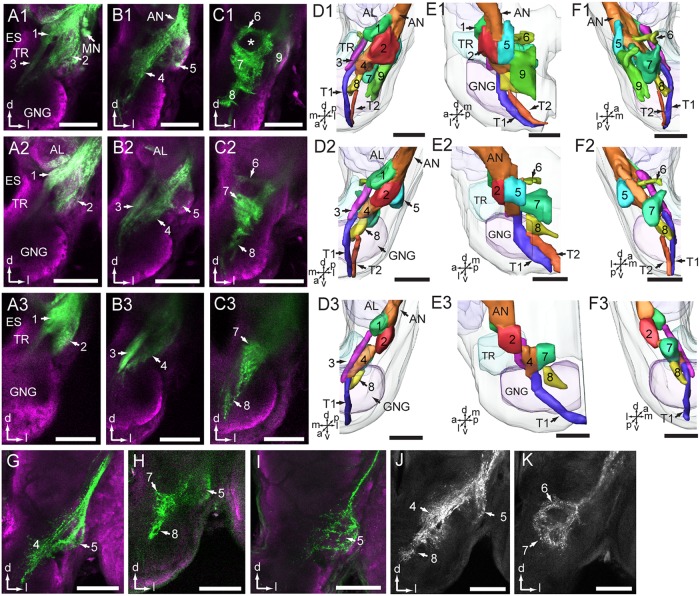
Projections of antennal sensory neurons in the antennal mechanosensory and motor center (AMMC). **(A1–C1)** Confocal images of antennal sensory projections in the AMMC, stained from the base of the scape. **(D1–F1)** Three-dimensional reconstructions of the sensory projections shown in **(A1–C1)**. Frontal view **(D1)**, lateral view **(E1)**, and posterior view **(F1)**. **(A2–C2)** Confocal images of antennal sensory projections in the AMMC, stained from the base of the pedicel. **(D2–F2)** Three-dimensional reconstruction of the sensory projections shown in **(A2–C2)**. Frontal view **(D2)**, lateral view **(E2)**, and posterior view **(F2)**. **(A3–C3)** Confocal images of antennal sensory projections in the AMMC, stained from the base of the flagellum. **(D3–F3)** Three-dimensional reconstruction of the sensory projections shown in **(A3–C3)**. Frontal view **(D3)**, lateral view **(E3)**, and posterior view **(F3)**. **(G,H)** Neuron projections of selectively stained Böhm bristles located on the scape. **(I)** Neuron projections of selectively stained Böhm bristles located on the pedicel. **(J,K)** Central projections of Johnston’s organ neurons. The numbers 1–8 in **(D,E, F)** indicate zones of the AMMC innervated by sub-groups of sensory axons. Number 9 indicates an area innervated by motor neurons. T1 and T2 indicate the descending tracts of antennal sensory neurons projecting to the ventral nerve cord. AL, antennal lobe; AN, antennal nerve; ES, esophagus; GNG, gnathal ganglion; MN, motor neuron; TR, tritocerebrum. a, anterior; d, dorsal; l, lateral; m, medial; p, posterior; v, ventral. Scale bars, 100 μm.

A substantial bundle of the antennal axons bypasses the AL on its lateral side and targets the AMMC (**Figure 4** and **Table 1**). According to the pattern of axon terminals, at least eight zones were identified, of which Zones 3, 4, and 8 partly overlap with the gnathal ganglion (**Figures 4A–F**). Zone 1 is located ventro-posteriorly of the AL, and Zone 2 ventrally to Zone 1 (**Figures 4A,D**). Zone 3, which is located laterally of the tritocerebrum, close to the dorsal midline of the gnathal ganglion, receives projections of antennal axons bypassing Zone 1 (**Figures 4A,D**). Zone 4 is located medio-posteriorly to the ventral part of Zone 2 and receives projections of axons bypassing Zone 1 (**Figures 4B,D**). Zone 5 is located posteriorly of Zone 2 (**Figures 4B,E**), Zone 6 medio-posteriorly of the dorsal AMMC, and Zone 7 medio-posteriorly of the ventral AMMC (**Figures 4C,F**). Zone 8, which borders with the midline of the gnathal ganglion and receives projections bypassing Zone 7, is located ventro-medially of Zones 4 and 7 (**Figures 4C,D**).

The preparations stained at the base of the scape and the pedicel showed similar projections patterns including labeled axons terminating in all eight zones of the AMMC (**Figures 4A1–F1,A2–F2**). The preparations stained at the base of the flagellum, on the other hand, showed labeled axons terminating in only six zones, i.e., Zones 1 – 4, plus 7 and 8 (**Figures 4A3-F3**).

Specific staining of Böhm bristles demonstrated that their sensory axons innervate almost the same areas, i.e., Zones 4, 5, 7, and 8, whereas specific staining of sensilla on the Johnstons’ organ gave rise to labeled terminals in Zones 4 – 8 (**Figures 4G–K** and **Table 1**). The preparations stained from the base of the scape showed a few labeled somata as well (**Figure 4A1**). According to previous reports from *Ma. sexta*, these are probably motor neurons (Kloppenburg et al., 1997). As demonstrated in **Figures 4D1–F1**, these neurons arborize mainly in a region located ventro-posteriorly in the AMMC (Zone 9). Notably, none of these motor neurons were stained when dye was applied at the base of the pedicel and the flagellum.

### Projection Pattern of Antennal Neurons in the Thoracic Ganglia

Within the axon bundle bypassing the AL, there are some long fibers forming two descending tracts that project into the ventral nerve cord. These paths, named T1 and T2, are separated when they bypass Zone 4 and 8, but fuse before entering the ventral nerve cord (**Figures 4D–F**).

**FIGURE 5:**
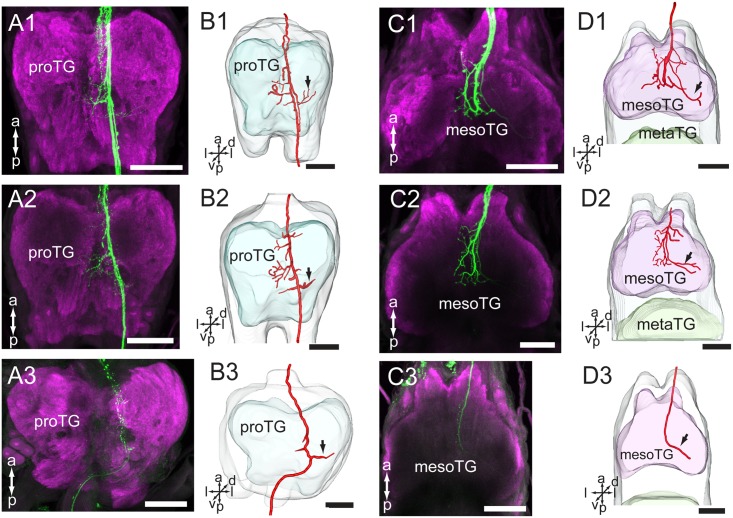
Projections of the antennal sensory neurons in the thoracic ganglia (TG) of the ventral nerve cord. **(A)** Confocal images of axon projections (green) in the proTG (magenta). **(B)** Three-dimensional reconstructions of the sensory axons shown in **(A)**. **(C)** Confocal images of axon projections (green) in the mesoTG (magenta). **(D)** Three-dimensional reconstruction of the sensory axons shown in **(C)**. In **(A1–A3)**, dye was applied at the base of the scape, pedicel and flagellum, respectively. In **(C1–C3)**, dye was applied at the base of the scape, pedicel and flagellum, respectively. Arrow indicates the long processes extending to the lateral part of the ganglion. mesoTG, mesothoracic ganglion; proTG: prothoracic ganglion. a, anterior; d, dorsal; l, lateral; p, posterior; v, ventral. Scale bars, 100 μm.

In the ventral nerve cord, the antennal axons project via the ipsilateral connective, first to the prothoracic ganglion (proTG) and then to the mesothoracic ganglion (mesoTG, **Figure 5** and **Table 1**). In the proTG, the axons pass near the midline and give off processes in the central area of the neuropil (**Figure 5A**). A few processes cross the midline to the contralateral side and some project to the lateral part of the neuropil (**Figure 5B**, indicated by arrows). The axons project further to the central area of the mesoTG (**Figure 5C**). Here, the axons make a loop and at the same time give off processes. As in the proTG, a few processes cross the midline to the contralateral side and some project to the lateral part of the neuropil (**Figure 5D**, indicated by arrows).

The preparations stained at the base of the scape and the pedicel showed similar projection patterns in the ventral nerve cord (**Figures 5A1–D1,A2–D2**), whereas those stained at the base of flagellum showed labeling of some axons of the T1 tract only. This fiber bundle, which is substantially thinner than the whole T1 tract, can be observed as it exits the gnathal ganglion and projects to the ventral nerve cord (**Figures 4D3–F3**, **5A3–D3**).

### Central Projections of the LPO Sensory Neurons

**FIGURE 6:**
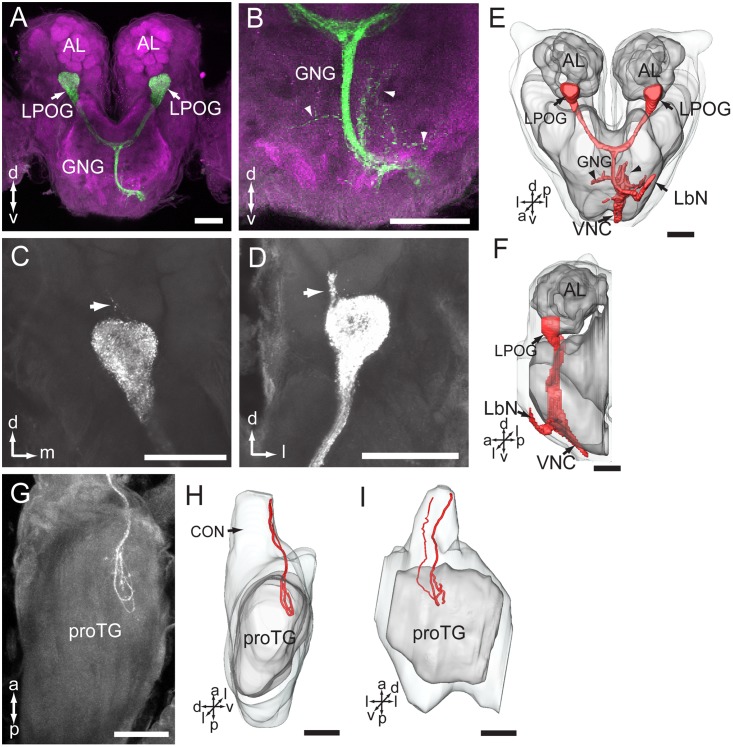
Central pathway of sensory neurons located in the labial palp organ (LPO). **(A)** Confocal image showing the main target region of the LPO sensory neurons (green), i.e., the LPO glomerulus (LPOG) in each antennal lobe (AL; magenta). **(B)** Enlarged image showing the sensory processes in the gnathal ganglion (GNG; arrowheads). **(C,D)** Confocal images showing terminals of stained axons innervating the entire LPOG, with a few processes outside of LPOG (arrows). **(E,F)** Three-dimensional reconstructions of the sensory pathway shown in **(A,B)**. **(E)** In frontal view, **(F)** in lateral view. **(G)** Confocal image showing axon projections in the prothoracic ganglion (TG). **(H,I)** Three-dimensional reconstructions of the axon terminals shown in **(G)**. **(H)** In lateral view, **(I)** in ventral view. CON, connective; LbN, labial nerve; VNC, ventral nerve cord. a, anterior; d, dorsal; l, lateral; p, posterior; v, ventral. Scale bars, 100 μm.

Among 33 preparations attempted labeled by applying dye into the LPO, 16 were successful. The stained axons of the LPO sensory neurons projected via the ipsilateral labial palp nerve and terminated in three main areas of the central nervous system: (1) LPOG in each AL, (2) the gnathal ganglion, and (3) the proTG of the ventral nerve cord (**Figure 6**). The most prominent target region was the LPOG. After entering the gnathal ganglion, a substantial portion of the stained axons divided into two bundles, the antenno-gnathal tracts, each projecting to the LPOG in one AL (**Figures 6A,E,F**). Here, the stained terminals filled the whole LPOG, and a few processes even extended outside the glomerulus (**Figures 6C,D**, indicated by arrows). Staining of antennal sensory neurons demonstrated that the LPOG does not receive any input from this neuronal category (**Figure 3B**, indicated by an asterisk). The second target region of LPO axons, the gnathal ganglion, is innervated by processes terminating mainly in the ipsilateral neuropil. Only a few stained branches extended to the contralateral side (**Figures 6B,E**, arrowheads). The third target region, the proTG of the ventral nerve cord, was innervated by axons leaving the gnathal ganglion via the ipsilateral connective (**Figures 6G–I**). In the proTG, the stained axons terminated in the anterior part of the neuropil forming a loop (**Figure 6G**). Males and females showed identical staining patterns in all three regions of the central nervous system.

## Discussion

### Antennal Sensory Neurons

Data obtained by electron microscopy showed that a large number of sensilla, including different morphological categories, are present on the flagellum of *My. separata*, whereas the pedicel and scape carry mainly Böhm bristles. These data are in full agreement with previous reports on the same species (Chang et al., 2015) and other insects (Schneider, 1964; Keil, 1999). The retrograde staining experiments visualized the antennal sensory neurons housed by the sensilla and their axons forming the AN. In addition, a large number of JONs located close to the inner surface of the pedicel, plus a few bipolar neurons at the inner surface of the scape were observed. Since no obvious sensilla were found on the external surface of the scape, these bipolar neurons may act as proprioceptors for the scape. Their specific function remains to be explored, however. Interestingly, the Böhm bristles and JONs are similar to those of *Ma. sexta*, which are found to work as mechanosensory neurons detecting movement and position of the antenna (Sane et al., 2007; Krishnan et al., 2012; Dieudonné et al., 2014). Thus, they are probably involved in flight control (Sane et al., 2007). The antennae of the monarch butterfly possess magnetic sensors and a circadian clock facilitating optimal orientation during migration (Merlin et al., 2009; Guerra et al., 2014). *My. separata* might actually possess corresponding arrangements as well, since it is reported to maintain consistent directions when migrating (Xu et al., 2017). Generally, the antennal sensilla of *My. separata* comprise several morphological types designed to detect different modalities, which in turn allow *My. separata* to perceive simultaneous input about the complex environment.

### Projection Pattern of the Antennal Sensory Neurons in the Central Nervous System

By combining electron microscopy with different mass staining experiments, including dye applied to the base of the scape, pedicel, and flagellum, respectively, we obtained a general overview of all antennal projections in the central nervous system as well as more detailed maps visualizing the target regions of sensory axons housed inside sensillum types located on the different antennal segments. The general staining pattern of all antennal afferents covered innervations in several ipsilateral regions of the central nervous system including the AL, protocerebrum, AMMC, gnathal ganglion, proTG, and mesoTG. Such a widespread projection pattern of the antennal axons was also observed in the locust, *Locusta migratoria*, blowfly, *Calliphora erythrocephala*, blood-sucking bug, *Rhodnius prolixus*, and mirid bug, *Apolygus lucorum* (Bräunig et al., 1983; Nässel et al., 1984; Barrozo et al., 2009; Xie et al., 2016). The multiple targeting areas may correspond to the different sensory modalities being detected by the antennal sensory neurons. Such a multisensory organ as the antennae of *My. separata* provides the flying insect with detailed information about the external world.

In addition to the afferent axons, a putative centrifugal neuron innervating the antenna was stained as well. This kind of neuron has not been reported in any insect species so far.

#### The AL and the Protocerebrum

Among all innervated regions, the AL was the most heavily stained area. This indicates a key role of the antenna in olfaction. The innervating pattern in the AL was similar in all three types of anterograde mass staining experiments, suggesting that the AL receives input from flagellar sensory neurons. In addition, specific staining of selected s. trichoidea showed labeling in AL glomeruli only, indicating that sensory neurons housed inside this numerous population of antennal sensilla terminate in the AL glomeruli exclusively. Such a projection pattern of olfactory sensory neurons has been found in many other insect species within a wide range of orders, for instance, dragonfly *Libellula depressa* (Rebora et al., 2013), cricket *Gryllus bimaculatus* (Yoritsune and Aonuma, 2012), cockroach *Periplaneta americana* (Nishino et al., 2005), louse *Columbicola columbae* (Crespo and Vickers, 2012), bugs *Euschistus heros*, *R. prolixus*, and *Apolygus lucorum* (Kristoffersen et al., 2008; Barrozo et al., 2009; Xie et al., 2016), aphid *Sitobion avenae*, *Metopolophium dirhodum*, and *Acyrthosiphon pisum* (Kristoffersen et al., 2008; Kollmann et al., 2011), psyllid *Trioza apicalis* (Kristoffersen et al., 2008), beetle *Tribolium castaneum* (Dippel et al., 2016), *A. mellifera* (Nishino et al., 2009), moth *Helicoverpa armigera* (Zhao et al., 2016), *D. melanogaster* (Stocker et al., 1983), *Aedes aegypti*, and *Anopheles gambiae* (Distler and Boeckh, 1997; Anton et al., 2003; Ignell et al., 2005).

A few stained axons projected to a region in the lateral protocerebrum. Similarly to the axons targeting the AL, they seem to originate from the flagellum. Projections of antennal sensory neurons targeting the protocerebrum were also observed in other insects, for instance, *L. depressa*, *Apolygus lucorum*, *C. erythrocephala*, *D. melanogaste*, *A. mellifera*, *Ae. aegypti*, and *An. gambiae* (Nässel et al., 1984; Ignell et al., 2005; Kamikouchi et al., 2006; Ai et al., 2007; Nishino et al., 2009; Rebora et al., 2013; Xie et al., 2016). However, these are mechanosensory neurons originating from the pedicel: in *C. erythrocephala* from the campaniform sensilla and in *D. menlanogaster* and *A. mellifera* from the Johnston’s organ (Nässel et al., 1984; Kamikouchi et al., 2006; Ai et al., 2007). In *My. separata*, on the other hand, the type of sensilla housing these neurons has not yet been identified.

#### The AMMC

The AMMC was the second most heavily stained area in the brain of *My. separata*. As its name suggests, the AMMC is the center for input from antennal mechanosensory neurons and for controlling movement of the antenna. Similarly to the general arrangement in the AL, the projection pattern of antennal sensory neurons in the AMMC is to a large extent conserved across a wide range of insects (Ignell et al., 2005; Nishino et al., 2005; Kamikouchi et al., 2006; Kristoffersen et al., 2008; Barrozo et al., 2009; Nishino et al., 2009; Kollmann et al., 2011; Crespo and Vickers, 2012; Yoritsune and Aonuma, 2012; Rebora et al., 2013; Xie et al., 2016; Zhao et al., 2016).

Altogether, the antennal sensory axons showed terminal projection into eight zones of AMMC of *My. separata*. However, the more detailed staining patterns in the AMMC differed when dye was applied to the base of the flagellum versus scape and pedicel. The fact that flagellar axons innervated Zones 1 – 4, plus 7 and 8, axons of Böhm bristles, located on the pedicel and scape, targeted Zones 4, 5, 7, and 8, whereas axons of JON terminated in Zones 4 – 8, suggests that distinct zones receive different signal categories.

Previous studies on the moths *Heliothis virescens* and *S. littoralis* including specific staining of s. chaetica have shown that their sensory neurons project to the AMMC and gnathal ganglion (Jørgensen et al., 2006; Popescu et al., 2013). The s. chaetica, situated on the flagellum, are contact chemosensors of moths, each housing four gustatory and one mechanosensory neuron (Jørgensen et al., 2006; Popescu et al., 2013). The different neurons project to distinct areas (Popescu et al., 2013). Their target areas in the AMMC seem to correspond to Zones 1 – 3 of *My. separata*.

The finding that sensory neurons of Böhm bristles, situated on the pedicel and scape, project to the same areas corresponds to previous reports from *Ma. sexta*. The previous studies showed that these neurons are involved in control of flight (Sane et al., 2007; Krishnan et al., 2012; Dieudonné et al., 2014). Concerning the JONs, it has been found, from investigations on *D. melanogaster* and *A. mellifera*, that these neurons also project to spatially segregated zones, and that the projection pattern is related to the locations of cell bodies (Kamikouchi et al., 2006; Ai et al., 2007). In *D. melanogaster*, it has been demonstrated that two distinct zones (named A and B) of the AMMC are responsible for near-field sound, and two others (named C and E) for gravitational forces and wind-induced deflections (Kamikouchi et al., 2009; Yorozu et al., 2009).

#### The Thoracic Ganglia

The fact that the projection pattern in the two ganglia was similar when staining from the scape and pedicel, and slightly reduced when staining from the flagellum, indicates that the labeled axons in the ventral nerve cord originate from the flagellum and pedicel exclusively. The general projection pattern of antennal afferents terminating in the proTG and mesoTG is similar to that observed in other insect species (Bräunig et al., 1983; Nässel et al., 1984; Barrozo et al., 2009; Xie et al., 2016). In particular, some of the sensory neurons originating from the flagellum and pedicel of *My. separata* extend a few long processes to lateral parts of the neuropils, known to be motor centers of the thorax (Nässel et al., 1984). This particular pattern is comparable with sensory neurons originating from campaniform sensilla on the pedicel in *C. erythrocephala* (Nässel et al., 1984). Interestingly, the axons of these neurons give off long bilateral branches into regions of the thoracic ganglia that act as leg motor centers (Nässel et al., 1984). Such an arrangement of antennal sensory neurons indicates a relatively close connection between the periphery and central motor areas presumably facilitating control of locomotion. The sensillum type housing the long antennal projections of *My. separata* has not yet been identified. The flagellar sensory neurons previously described in *Heliothis virescens* and *S. littoralis* were housed by s. chaetica (Jørgensen et al., 2006; Popescu et al., 2013). Since none of these neurons sent projections to the ventral nerve cord, it seems as if the descending sensory neurons of the flagellum originate from other sensillum types than s. chaetica.

### Projection Pathway of the Sensory Neurons of LPO in the Central Nervous System

The finding of stained LPO axons entering the ipsilateral side of the gnathal ganglion via the labial nerve and projecting to three distinct areas, the LPOG, gnathal ganglion, and proTG, is similar to the projection pattern previously reported in *Helicoverpa armigera* and other species (Bogner et al., 1986; Kent et al., 1986; Zhao et al., 2013). Several studies on moths have demonstrated that the sensory neurons in the LPO respond to CO_2_ (Bogner, 1990; Stange, 1992; Stange et al., 1995; Guerenstein et al., 2004). It is therefore reasonable to assume that the LPO neurons of *My. separata* are involved in CO_2_ detection as well. In particular, it would be interesting to investigate whether this kind of signaling might be involved in the long-distance migration of *My. separata*, taking place at altitudes of 200 – 500 m (Zhang et al., 2013).

The staining pattern in the LPOG of *My. separata*, including terminals from LPO axons filling the whole glomerulus, and no antennal axons, is in full agreement with previous reports in other moth species (Kent et al., 1986; Bogner, 1990; Zhao et al., 2013). Correspondingly, in the mosquitos, *Ae. aegypti*, and *An. gambiae*, CO_2_ sensitive neurons located on the distal segment of the maxillary palps project to AL glomeruli that receive no input from antennal sensory neurons (Distler and Boeckh, 1997; Anton et al., 2003). The finding of a few processes extending outside of LPOG in *My. separata* is not reported in any of the other studied species.

As mentioned above, the target regions of LPO projections in the gnathal ganglion and proTG of *My. separata*, were also observed in *Helicoverpa armigera* (Zhao et al., 2013). Also, previous findings from these two moth species have reported two morphologically corresponding types of LPO sensilla, one hair-shaped and one club-shaped (Zhao et al., 2013; Dong et al., 2014). In future studies, it would be interesting to map the projection pattern of axons originating from each sensillum category.

## Conclusion

The results of the present study show that the axons projecting from two sensory appendages of the moth *My. separata*, i.e., the antenna and the LPO, target multiple neuropils in the central nervous system including the protocerebrum, AL, AMMC, gnathal ganglion, and thoracic ganglia. These findings suggest that the antennae and LPOs play multiple roles in mediating insect behaviors. The AL is the major target for sensory axons originating from both sensory appendages. Furthermore, the AMMC, which is the second major target area for the antennal axons, receives no input from the LPO. The spatial organization of the terminals into segregated zones, shown for the first time in moths, indicates that the function of the mechanosensory neurons differs. The finding of processes from both the antenna and LPO in the gnathal ganglion suggests their roles in detection of gustation-related cues. In the ventral nerve cord, the processes from the LPO sensory neurons terminate in the proTG only, while the antennal afferents target both the proTG and mesoTG. Taken together, the results presented here provide a map of central projections originating from two sensory appendages, which might contribute to further understanding of how sensory information is processed and integrated in the central nervous system. This approach is particularly relevant since *My. separata* is a polyphagous and migratory pest relying on olfactory cues to locate nectar as well as mates, and probably integrating the chemosensory input with signals from other modalities during long-distance flight behavior.

## Author Contributions

Study concept and design: B-WM and X-CZ. Acquisition of data: B-WM, X-CZ, G-YX, and Q-BT. Analysis and interpretation of data: B-WM and X-CZ. Drafting of the manuscript: X-CZ and BB. Final manuscript: X-CZ, BB, and G-RW. Obtained funding: X-CZ and G-RW.

## Conflict of Interest Statement

The authors declare that the research was conducted in the absence of any commercial or financial relationships that could be construed as a potential conflict of interest.

## References

[B1] Ai H. Nishino H. Itoh T. (2007). Topographic organization of sensory afferents of Johnston’s organ in the honeybee brain. *J. Comp. Neurol.* 502 1030–1046. 10.1002/cne.21341 17444491

[B2] Altner H. Loftus R. (1985). Ultrastructure and function of insect thermo- and hygroreceptors. *Annu. Rev. Entomol.* 30 273–295. 10.1146/annurev.en.30.010185.001421

[B3] Anton S. van Loon J. J. A. Meijerink J. Smid H. M. Takker M. Rospars J. P. (2003). Central projections of olfactory neurons from single antennal and palpal sensilla in mosquitoes. *Arthropod Struct. Dev.* 32 319–327. 10.1016/j.asd.2003.09.002 18089015

[B4] Barrozo R. Couton L. Lazzari C. R. Insausti T. Minoli S. A. Fresquet N. (2009). Antennal pathways in the central nervous system of a blook-sucking bug, *Rhodnius prolixus*. *Arthropod Struct. Dev.* 38 101–110. 10.1016/j.asd.2008.08.004 18809510

[B5] Bogner F. (1990). Sensory physiological investigation of carbon dioxide receptors in lepidoptera. *J. Insect Physiol.* 36 951–957. 10.1016/0022-1910(90)90083-R

[B6] Bogner F. Boppré M. Ernst K. D. Boeckh J. (1986). CO_2_ sensitive receptors on labial palps of *Rhodogastria* moths (Lepidoptera: Arctiidae): physiology, fine structure and central projection. *J. Comp. Physiol. A* 158 741–749. 10.1007/BF01324818 3090241

[B7] Bräunig P. Pflüger H. J. Hustert R. (1983). The specificity of central nervous projections of locust mechanoreceptors. *J. Comp. Neurol.* 218 197–207. 10.1002/cne.902180207 6886072

[B8] Chang X. Q. Zhang S. Lv L. Wang M. Q. (2015). Insight into the ultrastructure of antennal sensilla of *Mythimna separata* (Lepidoptera: Noctuidae). *J. Insect Sci.* 15 124. 10.1093/jises/iev103 26363060 PMC4672215

[B9] Chen R. L. Bao X. Z. Drake V. A. Farrow R. A. Wang S. Y. Sun Y. J. (1989). Radar observations of the spring migration into northeastern China of the oriental armyworm moth, *Mythimna separata*, and other insects. *Ecol. Entomol.* 14 149–162. 10.1111/j.1365-2311.1989.tb00765.x

[B10] Christensen T. A. Hildebrand J. G. (2002). Pheromonal and host-odor processing in the insect antennal lobe: how different? *Curr. Opin. Neurobiol.* 12 393–399. 10.1016/S0959-4388(02)00336-7 12139986

[B11] Crespo J. G. Vickers N. J. (2012). Antennal lobe organization in the slender pigeon louse, *Columbicola columbae* (Phthiraptera: Ischnocera). *Arthropod Struct. Dev.* 41 227–230. 10.1016/j.asd.2012.02.008 22406082

[B12] de Bruyne M. Foster K. Carlson J. R. (2001). Odor coding in the *Drosophila* antenna. *Neuron* 30 537–552. 10.1016/S0896-6273(01)00289-6 11395013

[B13] Dieudonné A. Dandiel T. L. Sane S. P. (2014). Encoding properties of the mechanosensory neurons in the Johnston’s organ of the hawk moth, *Manduca sexta*. *J. Exp. Biol.* 217 3045–3056. 10.1242/jeb.101568 24948632

[B14] Dippel S. Kollmann M. Oberhofer G. Montino A. Krala M. Rexer K. H. (2016). Morphological and transcriptomic analysis of a beetle chemosensory system reveals a gnathal olfactory center. *BMC Biol.* 14:90. 10.1186/s12915-016-0304-z 27751175 PMC5067906

[B15] Distler P. G. Boeckh J. (1997). Central projections of the maxillary and antennal nerves in the mosquito Aedes aegypti. *J. Exp. Biol.* 200 1873–1879. 9319784 10.1242/jeb.200.13.1873

[B16] Dong J. F. Liu H. Tang Q. B. Liu Y. Zhao X. C. Wang G. R. (2014). Morphology, type and distribution of the labial-palp pit organ and its sensilla in the oriental armyworm, *Mythimna separata* (Lepidoptera: Noctuidae). *Acta Entomol. Sin.* 57 681–687.

[B17] Enjin A. Zaharieva E. E. Frank D. D. Mansourian S. Suh G. S. B. Gallio M. (2016). Humidity sensing in *Drosophila*. *Curr. Biol.* 26 1352–1358. 10.1016/j.cub.2016.03.049 27161501 PMC5305172

[B18] Feng H. Q. Zhao X. C. Wu X. F. Wu B. Wu K. M. Cheng D. F. (2008). Autumn migration of *Mythimna separata* (Lepidoptera: Noctuidae) over the Bohai sea in Northern China. *Environ. Entomol.* 37 774–781. 10.1093/ee/37.3.774 18559184

[B19] Frank D. D. Enjin A. Jouandet G. C. Zaharieva E. E. Para A. Stensmyr M. C. (2017). Early integration of temperature and humidity stimuli in the *Drosophila* brain. *Curr. Biol.* 27 2381–2388. 10.1016/j.cub.2017.06.077 28736172 PMC5600489

[B20] Gao Q. Yuan B. Chess A. (2000). Convergent projections of the *Drosophila* olfactory neurons to specific glomeruli in the antennal lobe. *Nat. Neurosci.* 3 780–785. 10.1038/75753 10903570

[B21] Guerenstein P. G. Hildebrand J. G. (2008). Roles and effects of environmental carbon dioxide in insect life. *Annu. Rev. Entomol.* 53 161–178. 10.1146/annurev.ento.53.103106.093402 17803457

[B22] Guerenstein P. G. Yepez E. A. van Haren J. Williams D. G. Hildebrand J. G. (2004). Floral CO_2_ emission may indicate food abundance to nectar-feeding moths. *Naturwissenschaften* 91 329–333. 10.1007/s00114-004-0532-x 15257387

[B23] Guerra P. A. Gegear R. J. Reppert S. M. (2014). A magnetic compass aids monarch butterfly migration. *Nat. Commun.* 5 4164. 10.1038/ncomms5164 24960099 PMC4090716

[B24] He Y. Feng B. Guo Q. Du Y. (2017). Age influences the olfactory profiles of the migratory oriental armyworm *Mythimna separata* at the molecular level. *BMC Genomics* 18:32. 10.1186/s12864-016-3427-2 28056777 PMC5217624

[B25] Ignell R. Dekker T. Ghaninia M. Hansson B. S. (2005). Neuronal architecture of the mosquito deutocerebrum. *J. Comp. Neurol.* 493 207–240. 10.1002/cne.20800 16255032

[B26] Jiang Y. Y. Li C. G. Zeng J. Liu J. (2014). Population dynamics of the armyworm in China: a review of the past 60 years’ research. *Chin. J. Appl. Entomol.* 51 890–898.

[B27] Jørgensen K. Kvello P. Almaas T. J. Mustaparta H. (2006). Two closely located areas in the suboesophageal ganglion and the tritocerebrum receive projections of gustatory receptor neurons located on the antennae and the proboscis in the moth *Heliothis virescens*. *J. Comp. Neurol.* 496 121–134. 10.1002/cne.20908 16528726

[B28] Kamikouchi A. Inagaki H. K. Effertz T. Hendrich O. Fiala A. Göpfert M. C. (2009). The neural basis of *Drosophila* gravity-sensing and hearing. *Nature* 458 165–171. 10.1038/nature07810 19279630

[B29] Kamikouchi A. Shimada T. Ito K. (2006). Comprehensive classification of the auditory sensory projections in the brain of the fruit fly *Drosophila* melanogaster. *J. Comp. Neurol.* 499 317–356. 10.1002/cne.21075 16998934

[B30] Keil T. A. (1999). “Morphology and development of the peripheral olfactory organs,” in *Insect Olfaction*, ed. Hansson B. S. (Berlin: Springer), 5–47. 10.1007/978-3-662-07911-9_2

[B31] Kent K. S. Harrow I. D. Quartararo P. Hildebrand J. G. (1986). An accessory olfactory pathway in Lepidoptera: the labial pit organ and its central projections in *Manduca sexta* and certain other sphinx moths and silk moths. *Cell Tissue Res.* 245 237–245. 10.1007/BF00213927 3742559

[B32] Kloppenburg P. Camazine S. M. Sun X. J. Randolph P. Hildebrand J. G. (1997). Organization of the antennal motor system in the sphinx moth *Manduca sexta*. *Cell Tissue Res.* 287 425–433. 10.1007/s004410050767 8995213

[B33] Kollmann M. Minoli S. Bonhomme J. Homberg U. Schachtner J. Tagu D. (2011). Revisiting the anatomy of the central nervous system of a hemimetabolous model insect species: the pea aphid *Acyrthosiphon pisum*. *Cell Tissue Res.* 343 343–355. 10.1007/s00441-010-1099-9 21170552

[B34] Krishnan A. Prabhakar S. Sudarsan S. Sane S. P. (2012). The neural mechanisms of antennal positioning in flying moths. *J. Exp. Biol.* 215 3096–3105. 10.1242/jeb.071704 22660776

[B35] Krishnan A. Sane S. P. (2015). Antennal mechanosensors and their evolutionary antecedents. *Adv. Insect Physiol.* 49 59–99. 10.1016/bs.aiip.2015.06.003

[B36] Kristoffersen L. Hansson B. S. Anderbrant O. Larsson M. C. (2008). Aglomerular hemipteran antennal lobes—basic neuroanatomy of a small nose. *Chem. Senses* 33 771–778. 10.1093/chemse/bjn044 18653643

[B37] Lin C. S. (1990). *The Physiology and Ecology of the Oriental Armyworm.* Beijing: Peking University Press.

[B38] Merlin C. Gegear R. J. Reppert S. M. (2009). Antennal circadian clocks coordinate sun compass orientation in migratory monarch butterflies. *Science* 325 1700–1704. 10.1126/science.1176221 19779201 PMC2754321

[B39] Nässel D. R. Högmo O. Hallberg E. (1984). Antennal receptors in the blowfly *Calliphora erythrocephala*. I. The gigantic central projection of the pedicellar campaniform sensillum. *J. Morphol.* 180 159–169. 10.1002/jmor.1051800206 30016846

[B40] Nishikawa M. Yokohari F. Ishibashi T. (1995). Central projections of the antennal cold receptor neurons and hygroreceptor neurons of the cockroach *Periplaneta americana*. *J. Comp. Neurol.* 361 165–176. 10.1002/cne.903610113 8550877

[B41] Nishino H. Nishikawa M. Mizunami M. Yokohari F. (2009). Functional and topographic segregation of glomeruli revealed by local staining of antennal sensory neurons in the honeybee *Apis mellifera*. *J. Comp. Neurol.* 515 161–180. 10.1002/cne.22064 19412930

[B42] Nishino H. Nishikawa M. Yokohari F. Mizunami M. (2005). Dual, multilayered somatosensory maps formed by antennal tactile and contact chemosensory afferents in an insect brain. *J. Comp. Neurol.* 493 291–308. 10.1002/cne.20757 16255033

[B43] Popescu A. Couton L. Almaas T. J. Rospars J. P. Wright G. A. Marion-Poll F. (2013). Function and central projections of gustatory receptor neurons on the antenna of the noctuid moth *Spodoptera littoralis*. *J. Comp. Physiol. A* 199 403–416. 10.1007/s00359-013-0803-0 23440349

[B44] Rebora M. Dell’Otto A. Rybak J. Piersanti S. Gaino E. Hansson B. S. (2013). The antennal lobe of *Libellula depressa* (Odonata, Libellulidae). *Zoology* 116 205–214. 10.1016/j.zool.2013.04.001 23816255

[B45] Sane S. P. Dieudonné A. Willis M. A. Daniel T. L. (2007). Antennal mechanosensors mediate flight control in moths. *Science* 315 863–866. 10.1126/science.1133598 17290001

[B46] Schneider D. (1964). Insect antennae. *Annu. Rev. Entomol.* 9 103–122. 10.1146/annurev.en.09.010164.000535

[B47] Stange G. (1992). High resolution measurement of atmospheric carbon dioxide concentration changes by the labial palp organ of the moth *Heliothis armigera* (Lepidoptera: Noctuidae). *J. Comp. Physiol. A* 171 317–324. 10.1007/BF00223962

[B48] Stange G. (1997). Effects of changes in atmospheric carbon dioxide on the location of hosts by the moth, *Cactoblanstis cactorum*. *Oecologia* 110 539–545. 10.1007/s004420050192 28307247

[B49] Stange G. Monro J. Stowe S. Osmond C. B. (1995). The CO_2_ sense of the moth *Cactoblastis cactorum* and its probable role in the biological control of the CAM plant *Opuntia stricta*. *Oecologia* 102 341–352. 10.1007/BF00329801 28306845

[B50] Stocker R. F. Singh R. N. Schorderet M. Siddiqi O. (1983). Projection patterns of different types of antennal sensilla in the antennal glomeruli of *Drosophila* melanogaster. *Cell Tissue Res.* 232 237–248. 10.1007/BF00213783 6411344

[B51] Vosshall L. B. Wong A. B. Axel R. (2000). An olfactory sensory map in the fly brain. *Cell* 21 147–159. 10.1016/S0092-8674(00)00021-0 10943836

[B52] Xie G. Y. Zhao X. C. Ma B. W. Guo P. Li G. P. Feng H. Q. (2016). Central projection of antennal sensory neurons in the central nervous system of the mirid bug *Apolygus lucorum* (Meyer-Dür). *PLOS ONE* 11:e0160161. 10.1371/journal.pone.0160161 27478892 PMC4968828

[B53] Xu J. Pan W. Zhang Y. Li Y. Wan G. Chen F. (2017). Behavioral evidence for a magnetic sense in the oriental armyworm, *Mythimna separata*. *Biol. Open* 6 340–347. 10.1242/bio.022954 28126710 PMC5374402

[B54] Yoritsune A. Aonuma H. (2012). The anatomical pathways for antennal sensory information in the central nervous system of the cricket, *Gryllus bimaculatus*. *Invert. Neurosci.* 12 103–117. 10.1007/s10158-012-0137-6 22669572

[B55] Yorozu S. Wong A. Fischer B. J. Dankert H. Kernan M. J. Kamikouchi A. (2009). Distinct sensory representations of wind and near-field sound in the *Drosophila* brain. *Nature* 458 201–205. 10.1038/nature07843 19279637 PMC2755041

[B56] Zhang Y. H. Zhang Z. Li C. Jiang Y. Y. Zeng J. Cheng D. F. (2013). Seasonal migratory behavior of *Mythimna separata* (Lepidoptera: Noctuidae) in northeast China. *Acta Entomol. Sin.* 56 1418–1429.

[B57] Zhao X. C. Chen Q. Y. Guo P. Xie G. Y. Tang Q. B. Guo X. R. (2016). Glomerular identification in the antennal lobe of the male moth *Helicoverpa armigera*. *J. Comp. Neurol* 524 2993–3013. 10.1002/cne.24003 27018863

[B58] Zhao X. C. Feng H. G. Wu B. Wu X. F. Liu Z. F. Wu K. M. (2009). Does the onset of sexual maturation terminate the expression of migratory behavior in moths? A study of the oriental armyworm, *Mythimna separata*. *J. Insect Physiol.* 55 1039–1043. 10.1016/j.jinsphys.2009.07.007 19643107

[B59] Zhao X. C. Tang Q. B. Berg B. G. Liu Y. Wang Y. R. Yang F. M. (2013). Fine structure and primary sensory projections of sensilla located in the labial-palp pit organ of *Helicoverpa armigera* (Insecta). *Cell Tissue Res.* 353 399–408. 10.1007/s00441-013-1657-z 23736380

